# Glomerular filtration rate estimation using the Cockcroft-Gault and Modification of Diet in Renal Disease formulas for digoxin dose adjustment in patients with heart failure

**DOI:** 10.1080/03009730903191853

**Published:** 2009-09-07

**Authors:** Marta Vazquez-Hernandez, Lorena Bouzas, J. Carlos Tutor

**Affiliations:** ^1^Servicio Farmacia, Complejo Hospitalario de OurenseOurenseSpain; ^2^Unidad Monitorización Fármacos, Laboratorio Central, Hospital Clínico UniversitarioSantiago de CompostelaSpain

**Keywords:** Cockcroft-Gault, digoxin total clearance, glomerular filtration rate, Modification of Diet in Renal Disease

## Abstract

**Objective:**

The aim of this study was to compare the estimated glomerular filtration rate (GFR) using the Cockcroft-Gault and the 4-, 5-, and 6-variable Modification of Diet in Renal Disease (MDRD) formulas for digoxin dose adjustment.

**Methods:**

Steady-state serum digoxin concentrations were determined in 100 patients with heart failure and normal to severely impaired renal function. Total clearance (CL) and predicted average concentrations of digoxin were calculated using general pharmacokinetic principles.

**Results:**

The mean±SEM (median) estimated GFR values were 48.9±2.8 (46.5) mL/min/1.73 m^2^ using the Cockcroft-Gault formula, 61.4±3.6 (56.4) mL/min/1.73 m^2^ using the MDRD4 formula, 56.8±3.3 (52.1) mL/min/1.73 m^2^ using the MDRD5 formula, and 53.3±3.0 (48.7) mL/min/1.73 m^2^ using the MDRD6 formula, with high correlation coefficients between the estimates (*r*≥ 0.928, *P<*0.001). Significant correlations were found between the digoxin total CL and estimated GFR by the Cockcroft-Gault (*r*=0.649, *P <*0.001), MDRD4 (*r*=0.634, *P <*0.001), MDRD5 (*r*=0.635, *P<*0.001), and MDRD6 (*r*=0.652, *P <*0.001) formulas. A significant negative correlation of the digoxin total CL/GFR ratio with estimated GFR was obtained (*r*= −0.356, *P<*0.001), with a high variability for this ratio for GFR lower than 60 mL/min. Analogous correlation coefficients were found between the obtained and predicted digoxin concentrations calculated using the estimated GFR by the Cockcroft-Gault (*r*=0.628, *P <*0.001), MDRD4 (*r*=0.642, *P <*0.001), MDRD5 (*r*=0.650, *P <*0.001), and MDRD6 (*r*=0.665, *P <*0.001) formulas, with a wide dispersion between the values in all cases.

**Conclusion:**

For GFR lower than 60 mL/min, the high interindividual variation of the digoxin total CL found among patients with similar renal function is an important limiting factor in the prediction of digoxin dosage regimens.

Digoxin has been used in therapeutics for more than two centuries; however, considering its economic and clinical benefits and its easy availability throughout the world, it should not be considered a drug of the past, with current approved uses for treating atrial fibrillation, with or without heart failure, and heart failure, with or without systolic dysfunction (1). The accepted therapeutic range for serum digoxin has changed in the past few years, and while the trough level of 2.0 µg/L is still useful in helping with the diagnosis of toxicity, at present it seems clear that digoxin should be administered in a dose to reach serum levels between 0.5 and 1.2 µg/L (1,2). The toxicity of this cardiac glycoside is dose-dependent, and as a substantial fraction of the absorbed dose is cleared by the kidneys, its toxicity is often the result of impaired renal function (1).

Glomerular filtration rate (GFR) is considered as the best measurement of kidney function, and its determination is important for drug dosage adjustment (3,4). GFR is currently estimated in clinical practice using different formulas based on serum creatinine, and the Cockcroft-Gault equation (5) is the most commonly used in pharmacokinetic studies and in the guidance of drug dosing. In an attempt to provide a more accurate estimate of GFR, the data from the Modification of Diet in Renal Disease (MDRD) study have been analysed and MDRD equations derived (6,7). It has been suggested that in most cases the GFR estimates from the Cockcroft-Gault and MDRD equations fall within the same interval for drug dose adjustment (8); however, discordant results have been obtained in different comparison studies (9–16).

The aim of our study was to compare GFR estimation from serum creatinine using the Cockcroft-Gault and the 4-, 5-, and 6-variable MDRD formulas in relation to therapeutic digoxin monitoring in patients with heart failure and normal to severely impaired renal function.

## Patients and methods

A group of 100 patients (43 male, 57 female) with a mean age (±SEM) of 79.4±0.8 years (range 47–94 years) with cardiac insufficiency was studied. They were given digoxin orally in tablet form in doses that had not been changed for at least 20 days beforehand, of between 0.125 and 0.25 mg/24–48 h. The blood samples were taken once the distribution stage was complete 24–48 hours after the last dose and correspond to the trough steady-state digoxin concentrations. The study was carried out according to the good practice rules for investigation in humans of the Consellería de Sanidade (Regional Ministry of Health) of the Xunta de Galicia, Spain.

Serum digoxin concentrations were determined by fluorescence polarization immunoassay in an Abbott TDx analyser using reagents from Abbott Laboratories (Abbott Park, IL, USA). The determination of serum creatinine, urea, and albumin was carried out in an Advia 2400 Chemistry System (Siemens Health Care Diagnostics Inc., Newark, DE, USA). The estimated GFR values from serum creatinine were calculated in accordance with the Cockcroft-Gault (5), and the 4-variable (age, sex, race, and serum creatinine) (MDRD4), 5-variable (age, sex, race, and serum creatinine and urea) (MDRD5), and 6-variable (age, sex, race, and serum creatinine, urea, and albumin) (MDRD6) equations (6,7), using the National Kidney Foundation GFR calculator (17). Height in the elderly is difficult to measure accurately (9), and this fact would introduce a misleading factor in the body surface area (BSA) calculation; however, the estimated GFR values by the Cockcroft-Gault and MDRD formulas are, respectively, expressed in mL/min and mL/min/1.73 m^2^, and consequently, within the context of our study, it was necessary in some cases to adjust the Cockcroft-Gault GFR values for the BSA of 1.73 m^2^, by dividing the estimates by BSA and multiplying by 1.73 m^2^. Inversely, the estimated GFR values using the MDRD formulas were multiplied by the BSA and divided by 1.73 m^2^ for their expression in mL/min. In order to calculate the digoxin total clearance (CL) and average serum steady-state concentration (Css) in the group of patients with heart failure studied, the following equations were used (18):

$$\begin{array}{l} \text{CL (mL/min)}\\ \;\text{= (0.33 mL/kg/min) }\times \text{ (weight in kg)}\\ \;\text{+0.9 (GFR in mL/min)} \end{array}$$ (1)

Css = (S) (F) (dose/τ)/CL (2)

S corresponds to the active fraction of the administered form (1 for digoxin), F is the bioavailability (0.7 for tablets), and τ the dosing interval.

Statistical analysis was carried out using the StatGraphics Plus (v. 5.0) package. The Shapiro-Wilks method was used to check the distribution of data, and Pearson's correlation coefficient was used when the data had a Gaussian distribution; otherwise, Spearman's correlation coefficient was used. The regression analysis was carried out using the Passing-Bablock non-parametric method. The comparison of the estimated GFR values was also carried out using the difference plots of Eksborg (19). In accordance with the proposed validation criteria of analytical methods for the quantitative determination of drugs and their metabolites, the acceptance criterion for accuracy is a deviation of no more than 15% from the nominal value (20,21). The results were expressed as mean±SEM (median), and statistical significance was considered as *P <*0.05.

## Results

**Figure 1.**
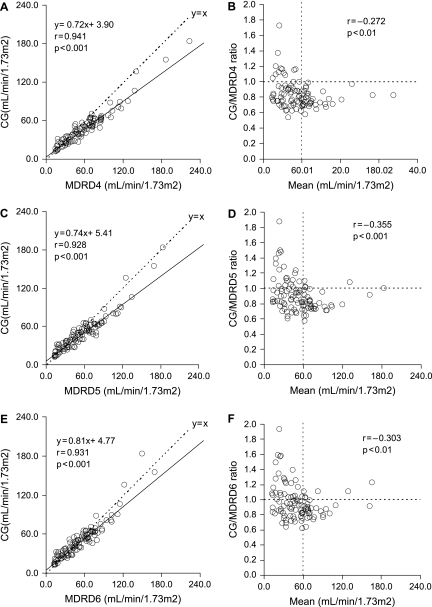
Correlation and regression (A, C, E) and Eksborg difference plots (B, D, F) between the estimated GFR values using the Cockcroft-Gault formula and those obtained using the MDRD4, MDRD5, and MDRD6 formulas.

In our group of patients, the estimated GFR values using the Cockcroft-Gault formula (48.9±2.8 (46.5) mL/min/1.73 m^2^) were significantly lower (*P <*0.001) than those obtained using the MDRD4 (61.4±3.6 (56.4) mL/min/1.73 m^2^), MDRD5 (56.8±3.3 (52.1) mL/min/1.73 m^2^), and MDRD6 (53.3±3.0 (48.7) mL/min/1.73 m^2^) formulas. Figure 1 shows the correlation and regression, and the difference plots, of the GFR values estimated by the Cockcroft-Gault formula with those obtained using the 4-, 5-, and 6-variable MDRD equations.

**Figure 2.**
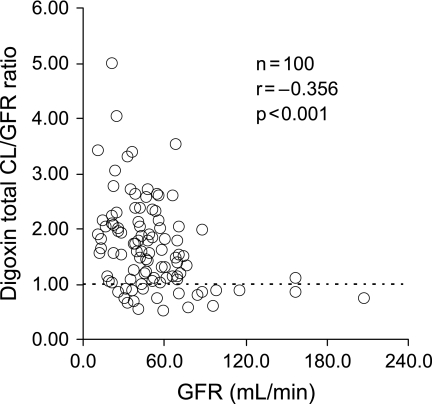
Relationship between the digoxin total CL/GFR ratio and GFR estimated by the Cockcroft-Gault formula.

Significant negative correlations were found between the digoxin total CL calculated from the obtained serum digoxin concentration using Equation 2, and the serum creatinine (*r*= − 0.594, *P <*0.001) and urea (*r*= − 0.547, *P <*0.001) concentrations. Analogous correlation coefficients were found between the digoxin total CL and the GFR values estimated by the Cockcroft-Gault (*r*=0.649, *P <*0.001), MDRD4 (*r*=0.634, *P <*0.001), MDRD5 (*r*=0.635, *P <*0.001), and MDRD6 (*r*=0.652, *P <*0.001) formulas. Significant negative correlations were obtained between the digoxin total CL/GFR ratio and the GFR estimated by the Cockcroft-Gault (Figure 2) or MDRD formulas (data not shown). As the obtained trough rather than average digoxin concentrations were used for its calculation using Equation 2, the digoxin total CL values considered in Figure 2 represent over-estimates of the actual values.

**Figure 3.**
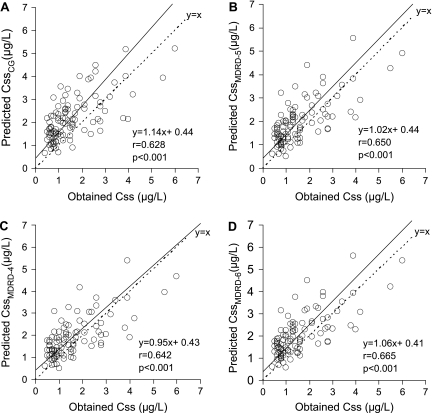
Correlation and regression between the obtained digoxin concentrations and those predicted calculated using the GFR estimated by the Cockcroft-Gault (A), MDRD4 (C), MDRD5 (B), and MDRD6 (D) formulas.

In the group of patients studied, the obtained serum (trough) concentration of digoxin (1.58±0.11 (1.25) µg/L) was significantly lower (*P <*0.001), with a deviation of more than 15%, than the digoxin (average) concentrations predicted by Equation 2, in where the digoxin total CL was calculated by means of Equation 1 and using the GFR estimates of the Cockcroft-Gault (2.17±0.10 (2.04) µg/L), MDRD4 (1.89±0.09 (1.74) µg/L), MDRD5 (1.99±0.09 (1.79) µg/L), and MDRD6 (2.06±0.10 (1.84) µg/L) formulas. Only the mean (median) predicted serum digoxin concentrations using the GFR values estimated by the Cockcroft-Gault and MDRD4 formulas have a deviation of more than 15% between them. Figure 3 shows the correlation and regression found between the obtained and predicted digoxin concentrations.

## Discussion

A substantial fraction of the absorbed digoxin is eliminated by the kidneys, with a renal CL approximately equal to or slightly lower than creatinine CL. In healthy persons the metabolic CL of digoxin is approximately 0.80 mL/min/kg, and congestive heart failure reduces this CL to around one-half its normal value, thereby slightly reducing the renal CL of the drug (18).

The GFR in the elderly, which in fact is prevalent in digoxin-treated patients, remains an unresolved problem as no equation has been validated in this population (9,13) and accurate measurements are rarely applicable in normal clinical settings. Different authors have stated that the Cockcroft-Gault formula cannot easily be replaced by the MDRD4 formula, which may over-estimate the GFR values resulting in different drug dosing recommendations (10,11,13–16). At present, the US Food and Drug Administration and the Kidney Disease Outcomes Quality Initiative of the National Kidney Foundation still recommend the use of the Cockcroft-Gault rather than the abbreviated MDRD formula for drug dose adjustment (15).

In our group of patients, high correlation coefficients (*r*≥0.928) were found between the GFR values estimated by the different formulas (Figure 1 A, C, E). In accordance with previously published data (10,11,13–16), the mean (median) GFR value estimated by the Cockcroft-Gault formula was significantly lower than those obtained using the MDRD4, MDRD5, and MDRD6 formulas (*P <*0.001). However, as indicated by the difference plots of Figure 1 (B, D, F), for GFR values lower than 60 mL/min/1.73 m^2^, the Cockcroft-Gault GFR estimates were frequently higher than those of the 4-, 5-, and 6-variable MDRD formulas.

It has recently been suggested that the 6-variable MDRD performs better than the Cockcroft-Gault formula in predicting aminoglycoside CL and may be considered as a tool for aminoglycoside-dosing recommendations (12). The MDRD4 equation tends to over-estimate and the Cockcroft-Gault formula to under-estimate in subjects aged 65 or older, and true GFR values could be situated between these two approximate values (9). The MDRD6 formula may comply with this requirement and would lead to a more accurate GFR estimation.

Contrary to O'Riordan et al. in healthy volunteers (22), in our patients with normal to severely impaired renal function we found a significant negative correlation of the digoxin total CL with serum creatinine and urea concentrations (*P <*0.001). Analogous correlation coefficients were obtained between the digoxin total CL and estimated GFR using the Cockcroft-Gault or the MDRD formulas (*r*≥0.634, *P <*0.001). In line with previous studies (23), the results shown in Figure 2 demonstrate that the great interindividual variability of the digoxin total CL/GFR ratio for GFR estimates lower than approximately 60 mL/min is an important limiting factor in the prediction of digoxin dosage regimens. Although 80% of the digoxin dose is excreted unchanged into urine in patients with normal renal function, in cases with renal failure the relative contribution of the hepatic elimination is increased and may be estimated to be as high as 75% in haemodialysis patients (24). As a result, the increase of the interindividual variation of the digoxin total CL, observed when GFR is lower than 60 mL/min among patients with similar renal function, could be attributed to differences in its hepatic elimination, a process that may be affected by the possible inhibition of the digoxin hepatic uptake by uraemic toxins (25). In any event, the possible impact of endogenous or exogenous digoxin-like immunoreactive substances on the commercial immunoassays used in therapeutic digoxin monitoring may be considered (26).

Analogous correlation coefficients were found between the obtained and predicted digoxin concentrations calculated using the GFR estimated by the Cockcroft-Gault or MDRD formulas (Figure 3), and only the mean (median) of the predicted digoxin concentrations using the Cockcroft-Gault and MDRD4 formulas had a deviation of more than 15% between them. However, the wide dispersion found between predicted and obtained digoxin concentrations is the main limiting factor for the clinical application of this predictive model. With respect to other recently proposed predictive digoxin dosage regimens (27), analogous considerations would be made. Assuming that digoxin CL remains stable in a patient, a more realistic use of Equation 2 may be to predict the steady-state digoxin concentration that will be achieved at a particular dosage in relation to the concentration previously obtained for another dose (28).
